# Optic neuropathy diagnosis in the emergency room - retrospective observational study of the last 18 years

**DOI:** 10.1177/11206721231173005

**Published:** 2023-05-22

**Authors:** Daniel Cardoso, Sofia Bezerra, Ricardo Soares-dos-Reis, Maria José Sá, Joana Guimarães

**Affiliations:** 1 Faculty of Medicine of the University of Porto, Porto, Portugal; 2 Department of Neurology, Centro Hospitalar de São João (CHUSJ), Porto, Portugal; 3 i3S - Institute for Research and Innovation in Health, University of Porto, Porto, Portugal; 4 Faculty of Health Sciences, University Fernando Pessoa, Porto, Portugal; 5 Center for Drug Discovery and Innovative Medicines (MedInUP), University of Porto, Portugal

**Keywords:** Optic neuropathy, optic neuropathies, optic nerve disorders, emergency room, emergency department, diagnosis, diagnostic accuracy.

## Abstract

**Introduction:**

Optic neuropathies (ON), a broad spectrum of disorders of the optic nerve, are a frequent cause of visual loss, presenting either in isolation or associated to neurological or systemic disorders. They are often first evaluated in the Emergency Room (ER) and a rapid determination of the etiology is imperative for implementing timely and appropriate treatment. We aim to describe ER demographic data and clinical characteristics, as well as the performed imaging exams, of patients subsequently hospitalized and diagnosed with ON. Furthermore, we seek to explore the accuracy of ER discharge diagnosis and evaluate possible predictive factors that may influence it.

**Methods:**

We retrospectively reviewed the medical records of 192 patients admitted to the ward of the Neurology Department of Centro Hospitalar Universitário São João (CHUSJ), with a discharge diagnosis of ON. Subsequently, we selected those admitted from the ER, with clinical, laboratory and imaging data, between January 2004 and December 2021.

**Results:**

We included 171 patients. All participants were discharged from the ER and admitted in the ward with a main diagnostic suspicion of ON. Patients were stratified according to suspected etiology at the time of discharge: 99 inflammatory (57.9%), 38 ischemic (22.2%), 27 unspecified (15.8%) and 7 other (4.1%). By comparing with current follow-up diagnosis, 125 patients had an accurate ER diagnosis category (73.1%), 27 had an ON diagnosis of unspecified etiology that was defined only during follow-up (15.8%) and 19 had an inaccurate diagnosis category (11.1%). Diagnostic change was more common with ER ischemic diagnosis (21.1%) compared to inflammatory diagnosis (8.1%) (p = 0.034).

**Conclusions:**

Our study reveals that most patients with ON can be accurately diagnosed in the ER through clinical history neurological and ophthalmological evaluation.

## Introduction

Disorders of the optic nerves, known as “Optic neuropathies “(ON), are some of the most common causes of vision loss.^1,2^ The etiological causes are diverse, and ON can present isolated or as part of broader neurological or systemic disorders.

ON can often be first clinically observed in the Emergency Room (ER).^3^ Once that a major challenge is that the prognosis and the therapeutic strategy can differ according to the mechanism involved, the initial work-up is fundamental to determine the specific cause, efficient diagnostic assessment and appropriate therapeutic strategy.^1,2,4^

In what concerns the clinical history and examination, ON can present with diverse neuro-ophthalmological findings,and vision loss may be preceded or simultaneous to eye pain/discomfort.^5^ Since the optic nerve and retina extend from the developing brain in embryonic development, the evaluation of ON and the optic nerve makes them one of the best and least invasive methods for investigating central nervous system pathologies, in both clinical practice and scientific research.^1,6^

Failing to obtain a careful clinical history and detailed neuro-ophthalmologic examination in the ER can result in inadequate interventions with mild-to-severe vision impairment, as some ON are true medical emergencies.^7,8^

Accurate diagnosis is expected to rely mostly on the evaluation of key clinical signs for distinguishing ON from other pathologies,^4,8^ and medical history with physical examination (visual acuity, color vision, confrontation visual fields, pupillary and fundoscopic examination, in addition to a targeted neurological exam) for uncovering the specific etiology.^4^ Although the clinical diagnosis of ON can be usually reached with an adequate initial observation, determining the specific cause of the ON can represent a greater difficulty, particularly in the emergency care setting, making the diagnosis established *ab initio* potentially redefined after follow-up and/or further investigations.^9^

Currently, ON remains mostly a clinical diagnosis; nevertheless, advances in optic nerve imaging, as well as antibody testing, have gained growing importance in the diagnosis and follow-up of patients with ON,^1,2,10,11^ despite their possible risk of misdiagnosis.^11,12^

There is limited data and reports regarding the presentation and examination of optic neuropathies in the ER setting. We aim with this study to report ER demographic data, clinical characteristics and describe the performed imaging exams, of patients subsequently hospitalized and diagnosed with ON. Additionally, we seek to determine whether the initial diagnostic suspicion made by the ER neurologist was confirmed after definitive diagnosis. Furthermore, our study intends to identify possible predictive factors that may influence the diagnostic accuracy of ER observation. For this purpose, we reviewed the data of all patients seen in the ER in the past 18 years who were subsequently admitted to the neurology ward of Centro Hospitalar Universitário de São João (CHUSJ) and discharged with a diagnosis of ON.

## Methods

### Study design and setting

We performed a retrospective evaluation of demographic data and clinical characteristics,as well as of the performed imaging exams and follow-up in all patients discharged from the neurology ward of CHUSJ with a diagnosis of ON and previously seen in the ER. Data was collected from patients’ admission to the ER between 5th January 2004 and 17^th^ December 2021. Patient's information was retrieved retrospectively, until February 2022, from an electronic hospital-wide database. Patients’ follow-up data was researched until the date of their current diagnosis, mostly coinciding with the neurology ward discharge date. In order to address potential sources of bias we selected a clinically relevant population of substantial size and applied the same criteria for all participants. All patients were observed by neurologists and ophthalmologists in the ER before ward admission. Research was conducted in compliance with the Ethics Committee of CHUSJ and by the Data Protection Officer.

### Participants

We included all discharged patients from the neurology ward of CHUSJ with a diagnosis of ON (n = 192). Twenty-one patients were excluded: out of these, 13 for lack of ER data and 8 because they were admitted to the ward from medical consultation. Hence, this study's population comprises 171 patients.

### Diagnostic categories of optic neuropathies

We stratified initial ER diagnosis suspicion in 4 ON categories.^2,4^: Inflammatory: multiple sclerosis and other associated Inflammatory/demyelinating disorders; Ischemic: Anterior Ischemic Optic Neuropathy (AION), including Arteritic AION (AAION) and Nonarteritic AION (NAION), and Posterior Ischemic Optic Neuropathy (PION); Other etiologies: infectious, systemic vasculitis, hereditary and nutritional deficiency; Unspecified: inconclusive etiological diagnosis

We stratified the follow-up (current) diagnosis in 5 ON categories^6,10,12^: Multiple sclerosis; Other demyelinating/inflammatory – includes Neuromyelitis Optica Spectrum Disorder (NMOSD), MOG Antibody Disease (MOGAD) and Chronic Relapsing Inflammatory Optic Neuritis (CRION); Idiopathic Optic Neuritis; Ischemic ON - includes AAION and NAION; Other ON – includes toxic, nutritional, infectious, and systemic vasculitis ON.

We compared participants initial ER diagnosis category with follow-up category to determine diagnostic accuracy. Accurate diagnosis was considered between the following matching categories: Inflammatory with multiple sclerosis, other demyelinating/inflammatory ON or idiopathic optic neuritis; Ischemic with AAION or NAION; Other ON according to specific etiology.

### Variables

We retrieved data about clinical presentation at ER observation (including date, gender, age, interval between symptom onset and ER admission, affected eye(s), Best Corrected Visual Acuity (BCVA), presence of eye pain and other ophthalmic symptoms and onset, fundoscopic evaluation, relative afferent pupillary defect (RAPD), neurologic examination, past medical and family history) and diagnostic exams performed**.** The BCVA was examined by Snellen chart and converted into decimal values as required by European norm (EN ISO 8596). For statistical analysis, we classified as having visual acuity <0.1, patients with visual acuity described as “finger counting”, “hand motion”, “light perception” or “no light perception”. Orbit and Head Computed Tomography scan (CT scan) and Magnetic Resonance Imaging (MRI) performed in the ER and during follow-up were interpreted by radiologists. Retinal optical coherence tomography (OCT) was introduced in ER clinical practice for the diagnosis of ON in 2014.^11^ During neurology ward follow-up, OCT and Visual Evoked Response (VER) tests were used. Biochemical and immunological blood tests, cerebrospinal fluid tests as well as myelin oligodendrocyte glycoprotein IgG (MOG-IgG) at the start of 2018, and anti-aquaporin-4 IgG (AQP4-IgG) cell-based assays were performed as diagnostic tools during follow-up.^10,12^

### Statistical analysis

Statistical analysis was performed with SPSS Statistics, version 26. For categorical variables, absolute frequencies (n) and percentages (%) were calculated. For continuous variables, medians (Mdn) and percentiles (P25-P75) were calculated, after checking their non symmetrical distributions. Associations with categorical variables were assessed with chi-square tests or Fisher exact tests, when more than 20% of the cells had expected frequency lower than 5. In the case of significant associations detected with these tests, standardized residuals (SR) were calculated and presented to increase perception about the direction of associations. Missing data was omitted from analysis. Associations of continuous variables with categorical ones were assessed with Mann-Whitney tests (2 groups) and Kruskall-Wallis (3 or more groups). Significance level was considered for p < 0.05.

## Results

We reviewed the medical files of 192 patients discharged from the neurology ward of CHUSJ with a diagnosis of ON. Out of these, 184 (95.8%) were sent from the ER, while 8 (4.2%) came from a medical consultation: 3 from Demyelinating Diseases; 3 from General Neurology; 1 from Headache Neurology; 1 from Ophthalmology.

Out of the initial population, we excluded 13 for lack of ER data and 8 because they were admitted to the ward from a medical consultation. Of the remaining 171 patients included for statistical analysis, we found that all were discharged from the ER and admitted in the ward with a main diagnostic suspicion of ON. Patients were stratified according to suspected etiology at the time of discharge: 99 inflammatory (57.9%), 38 ischemic (22.2%), 27 unspecified (15.8%) and 7 other (4.1%). By comparing with current follow-up diagnosis, 125 patients had an accurate ER diagnosis category (73.1%), 27 had an initial ON diagnosis of unspecified etiology that was defined only during follow-up (15.8%) and 19 had an inaccurate diagnosis category (11.1%).

Our statistical analysis found that a diagnostic change was more common with initial ischemic diagnosis (21.1%) when comparing to inflammatory diagnosis (8.1%) (p = 0.034). Regarding this lower diagnostic accuracy, the analysis of our data reveals also that patients misdiagnosed as having ischemic ON had a greater prevalence of visual acuity > 0,1 (81.8% vs 31.6%, p = 0.005) and absence of previous neurological events (90.9% vs 55.3%, p = 0.038) compared to patients with actual Ischemic ON. The median age was also lower in the misdiagnosed population (53.0 vs 62.5 years) (p = 0.023). We also found that of the initial 34 patients with suspected unilateral Ischemic ON, only 4 patients (11.8%) had the contralateral eye examined for the presence of crowded disk.

Clinical features and exams performed in the ER according to diagnostic accuracy, are compiled in Table 1. Most patients in our cohort were women (62.6%), the mean age of disease onset was 42.5 years (40.9 for women; 45.2 for men), with 21 patients having bilateral eye disease at admission (12.3%). Regarding visual loss, 74 patients presented with vision acuity <0.1 (45.1%).

**Table 1.** Clinical features and exams performed in the emergency room according to accuracy of diagnosis of optic neuropathy category.

Emergency Room diagnostic accuracy for Optic Neuropathy category									
	Inaccurate		Accurate		Unspecified etiology		Total		
	n	% (SR)	n	% (SR)	n	% (SR)	n	%	p- value
Patient's gender									.420
Woman	12	63.2%	75	60.0%	20	74.1%	107	62.6%	
Man	7	36.8%	50	40.0%	7	25.9%	64	37.4%	
Eye affected									.062
Left	9	47.4%	56	44.8%	7	25.9%	72	42.1%	
Right	5	26.3%	57	45.6%	16	59.3%	78	45.6%	
Both	5	26.3%	12	9.6%	4	14.8%	21	12.3%	
Visual acuity - Affected Eye at admission									.220
≤0,1	5	27.8%	55	45.8%	14	53.8%	74	43.3%	
>0,1	13	72.2%	65	54.2%	12	46.2%	90	52.6%	
Presence of Scotoma									.099
No	12	63.2%	101	80.8%	24	88.9%	137	80.1%	
Yes	7	36.8%	24	19.2%	3	11.1%	34	19.9%	
Presence of Dyschromatopsia									.417
No	17	89.5%	95	76.0%	20	74.1%	132	77.2%	
Yes	2	10.5%	30	24.0%	7	25.9%	39	22.8%	
Presence of relative afferent pupillary defect									.458
No	2	10,5%	26	20,8%	5	18,5%	33	19.3%	
Yes	10	52.6%	72	57,6%	13	48.1%	95	55.6%	
Not performed	7	36.9%	27	21,6%	9	33,3%	43	25.1%	
Presence of superior altitudinal visual field defect									.670
No	18	94.7%	119	95.2%	27	100.0%	164	95.9%	
Yes	1	5.3%	6	4.8%	0	0.0%	7	4.1%	
Presence of inferior altitudinal visual field defect									.266
No	19	100.0%	109	87.2%	24	88.9%	152	88.9%	
Yes	0	0.0%	16	12.8%	3	11.1%	19	11.1%	
Onset of ophthalmic symptoms									.866
Progressive	9	50.0%	67	55.4%	14	56.0%	90	54.9%	
Sudden	3	16.7%	27	22.3%	6	24.0%	36	22.0%	
Waking up	6	33.3%	27	22.3%	5	20.0%	38	23.1%	
Eye Pain									.968
No	10	52.6%	59	47.6%	13	48.1%	82	47.9%	
Yes	9	47.4%	65	52.4%	14	51.9%	88	51.5%	
Fundus examination: optic disc edema									.175
Absence	7	36.8%	50	41.7%	6	22.2%	63	38.0%	
Presence	12	63.2%	70	58.3%	21	77.8%	103	62.0%	
Fundus examination: optic disc pallor									.864
Absence	16	84.2%	105	84.0%	24	88.9%	145	87.3%	
Presence	3	15.8%	15	12.5%	3	11.1%	21	12.7%	
Neurological examination: presence of neurologic alterations									>.990
No	18	94.7%	113	90.4%	25	92.6%	156	91.2%	
Yes	1	5.3%	12	9.6%	2	7.4%	15	8.8%	
Presence of other symptoms									.484
No	10	52.6%	78	62.4%	19	70.4%	107	62.6%	
Yes	9	47.4%	47	37.6%	8	29.6%	64	37.4%	
Previous neurological events									.216
No	12	63.2%	80	64.0%	22	81.5%	114	66.7%	
Yes	7	36.8%	45	36.0%	5	18.5%	57	33.3%	
Past medical history: autoimmune disease									.242
No	18	94.7%	113	90.4%	27	100.0%	158	92.4%	
Yes	1	5.3%	12	9.6%	0	0.0%	13	7.6%	
Past medical history: cardiovascular disease risk factors									.824
No	11	57.9%	64	51.2%	15	55.6%	90	52.6%	
Yes	8	42.1%	61	48.8%	12	44.4%	81	47.4%	
Family disease									.711
Irrelevant	14	77.8%	83	76.9%	20	74.1%	98	77.0%	
Neurologic disease	4	22.2%	23	21.3%	3	11.1%	27	21.4%	
Autoimmune disease	0	0.0%	2	1.9%	1	3.7%	2	1.6%	
Performed Orbit CT scan									.588
No	17	89.5%	102	81.6%	24	88.9%	143	83.6%	
Yes	2	10.5%	23	18.4%	3	11.1%	28	16.4%	
Performed Head CT scan									.476
No	4	21.1%	28	22.4%	3	11.1%	35	20.5%	
Yes	15	78.9%	97	77.6%	24	88.9%	136	79.5%	
Performed Brain MRI									>.990
No	19	100.0%	122	97.6%	27	100.0%	168	98.2%	
Yes	0	0.0%	3	2.4%	0	0.0%	3	1.8%	
Performed Optical Coherence Tomography (OCT)									.239
No	16	84.2%	117	93.6%	24	88.9%	157	91.8%	
Yes	3	15.8%	8	6.4%	3	11.1%	14	8.2%	

RAPD was evaluated in 128 patients of our population (74.8%) and it was found in 88 of these (74.2%). Of the remaining 43 patients that were not evaluated for RAPD, 4 patients were under use of topical mydriatic agent.

Regarding practitioner's use of imaging exams in the ER, out of the 171 patients, 136 performed a head CT scan (79%), 28 an orbit CT scan (16%), 14 an OCT (8.2% total, and 14.7% since OCT use for ON began in 2014) and 3 a brain MRI (1.8%). We found no statistically significative difference in diagnostic accuracy between patients who were submitted to imaging exams (CT scan, MRI, and OCT) in the ER and those who were not.

An overview of demographic and clinical ER characteristics of study participants, according to their diagnostic group at the end of follow-up, is represented in Table 2 **and **Table 3. A significant association was found between follow-up ON diagnosis and ER diagnostic accuracy (p < 0.001). Misdiagnosis was less prevalent in the multiple sclerosis group (2.3%, SR = −1.8) but overrepresented in the other ON group (54.5%, SR = 4.3). An ER optic neuropathy diagnosis of unspecified etiology was more frequent in the other demyelinating/inflammatory group (42.9%, SR = 2.5). Presence of scotoma was more frequently associated with other ON group (p = .045), with a 54.5% prevalence (SR = 2.6).

**Table 2.** Emergency room diagnosis and clinical presentation of patients according to optic neuropathy diagnostic group at the end of follow-up.

Optic Neuropathy diagnostic group at end of follow-up													
	Multiple sclerosis		Idiopathic optic neuritis		Other demyelinating/ inflammatory			Ischemic	Other Optic Neuropathy		Total		
	n	% (SR)	n	% (SR)	n	% (SR)	n	% (SR)	n	% (SR)	n	%	p-value
Emergency Room Diagnosis Category													
Inflammatory	39	88.6%	44	72.1%	8	57.1%	5	12.2%	3	27.3%	99	57.9%	-
Unspecified etiology	4	9.1%	10	16.4%	6	42.9%	6	14.6%	1	9.1%	27	15.8%	
Ischemic	1	2.3%	4	6.6%	0	0.0%	30	73.2%	3	27.3%	38	22.2%	
Other	0	0.0%	3	4.9%	0	0.0%	0	0.0%	4	36.4%	7	4.1%	
Emergency Room Diagnostic accuracy													<.001
Inaccurate	1	2.3% (−1.8)	7	11.5% (0.1)	0	0.0% (−1.2)	5	12.2% (0.2)	6	54.5% (4.3)	19	11.1%	
Accurate	39	88.6% (1.2)	44	72.1% (−0.1)	8	57.1% (−0.7)	30	73.2% (0.0)	4	36.4% (−1.4)	125	73.1%	
Unspecified etiology	4	9.1% (−1.1)	10	16.4% (0.1)	6	42.9% (2.5)	6	14.6% (−0.2)	1	9.1% (−0.6)	27	15.8%	
Patient's gender													.626
Woman	31	70.5%	37	60.7%	8	57.1%	23	56.1%	8	72.7%	107	62.6%	
Man	13	29.5%	24	39.3%	6	42.9%	18	43.9%	3	27.3%	64	37.4%	
Eye affected													.727
Left	21	47.7%	22	36.1%	6	42.9%	17	41.5%	6	54.5%	72	42.1%	
Right	18	40.9%	30	49.2%	8	57.1%	19	46.3%	3	27.3%	78	45.6%	
Both	5	11.4%	9	14.8%	0	0.0%	5	12.2%	2	18.2%	21	12.3%	
Visual acuity - Affected Eye													.963
≤0.1	19	44.2%	25	43.9%	7	53.8%	23	56.1%	5	50.0%	74	45.1%	
>0.1	24	55.8%	32	56.1%	6	46.2%	18	43.9%	5	50.0%	90	54.9%	
Presence of Scotoma													.045
No	34	77.3% (−0.2)	52	85.2% (0.4)	13	92.9% (0.5)	33	80.5% (0.0)	5	45.5% (−1.3)	137	80.1%	
Yes	10	22.7% (0.4)	9	14.8% (−0.9)	1	7.1% (−1.1)	8	19.5% (−0.1)	6	54.5% (2.6)	34	19.9%	
Presence of Dyschromatopsia													.388
No	31	70.5%	51	83.6%	9	64.3%	32	78.0%	9	81.8%	132	77.2%	
Yes	13	29.5%	10	16.4%	5	35.7%	9	22.0%	2	18.2%	39	22.8%	
Relative afferent pupillary defect													.667
No	7	15,9%	11	18,0%	4	28,6%	8	19,5%	3	27,3%	33	25,8%	
Yes	29	65,9%	31	50,8%	8	57,1%	23	56,1%	4	36,4%	95	74,2%	
Superior altitudinal visual field defect													.936
No	42	95.5%	59	96.7%	13	92.9%	39	95.1%	11	100.0%	164	95.9%	
Yes	2	4.5%	2	3.3%	1	7.1%	2	4.9%	0	0.0%	7	4.1%	
Inferior altitudinal visual field defect													.185
No	42	95.5%	55	90.2%	13	92.9%	33	80.5%	9	81.8%	152	88.9%	
Yes	2	4.5%	6	9.8%	1	7.1%	8	19.5%	2	18.2%	19	11.1%	
Onset of Ophthalmic Symptoms													.609
Progressive	22	51.2%	35	62.5%	7	50.0%	21	52.5%	5	45.5%	90	54.9%	
Sudden	11	25.6%	10	17.9%	5	35.7%	9	22.5%	1	9.1%	36	22.0%	
Waking up	10	23.3%	11	19.6%	2	14.3%	10	25.0%	5	45.5%	38	23.2%	
Eye Pain													.080
No	25	58.1%	24	42.1%	4	30.8%	25	61.0%	4	40.0%	82	48.2%	
Yes	19	44.2%	36	63.2%	10	76.9%	16	39.0%	7	70.0%	88	51.8%	
Fundus examination: optic disc edema													.358
Absence	22	50.0%	20	35.5%	4	28.6%	13	31.7%	4	44.4%	63	38.0%	
Presence	22	50.0%	38	65.5%	10	71.4%	28	68.3%	5	55.6%	103	62.0%	
Fundus examination: optic disc pallor													.434
Absence	40	90.9%	50	86.2%	10	71.4%	36	90.0%	9	90.0%	145	87.3%	
Presence	4	9.1%	8	13.8%	4	28.6%	4	10.0%	1	10.0%	21	12.7%	
Presence of neurologic alterations													.722
No	41	93.2%	54	88.5%	12	85.7%	38	92.7%	11	100.0%	156	91.2%	
Yes	3	6.8%	7	11.5%	2	14.3%	3	7.3%	0	0.0%	15	8.8%	
Presence of other symptoms													.058
No	32	72.7%	31	50.8%	12	85.7%	26	63.4%	6	54.5%	107	62.6%	
Yes	12	27.3%	30	49.2%	2	14.3%	15	36.6%	5	45.5%	64	37.4%	
Medical history: previous neurological event													.644
No	26	59.1%	40	65.6%	10	71.4%	29	70.7%	9	81.8%	114	66.7%	
Yes	18	40.9%	21	34.4%	4	28.6%	12	29.3%	2	18.2%	57	33.3%	
Medical history: autoimmune disease													.875
No	42	95.5%	55	90.2%	13	92.9%	38	92.7%	10	90.9%	158	92.4%	
Yes	2	4.5%	6	9.8%	1	7.1%	3	7.3%	1	9.1%	13	7.6%	
Medical history: cardiovascular risk factors													.988
No	23	52.3%	33	54.1%	7	50.0%	22	53.7%	5	45.5%	90	52.6%	
Yes	21	47.7%	28	45.9%	7	50.0%	19	46.3%	6	54.5%	81	47.4%	
Family disease													.740
Irrelevant	34	82.9%	43	78.2%	8	72.7%	25	75.8%	7	70.0%	117	78.0%	
Neurologic disease	7	17.1%	11	20.0%	2	18.2%	7	21.2%	3	30.0%	30	20.0%	
Autoimmune disease	0	0.0%	1	1.8%	1	9.1%	1	3.0%	0	0.0%	3	2.0%	

**Table 3.** Emergency room continuous clinical variables according to optic neuropathy diagnostic group at the end of follow-up.

	Multiple clerosis			Idiopathic optic neuritis			Other demyelinating/inflammatory			Ischemic			Other ON			
	Mdn	P25	P75	Mdn	P25	P75	Mdn	P25	P75	Mdn	P25	P75	Mdn	P25	P75	p-value
Age at admission	37.0	29.0	49.5	37.0	27.0	53.0	46.0	30.0	54.0	45.0	33.0	62.0	37.0	24.0	47.0	.313
Interval between symptom onset and admission at emergency room (days)	3.0	2.0	7.0	5.0	2.0	7.0	3.5	2.0	7.0	4.0	3.0	7.0	7.0	2.0	14.0	.666
Visual acuity - Affected Eye: (10/10)	2.0	0.0	5.0	2.0	0.0	5.0	1.0	0.0	8.0	3.0	0.0	7.0	1.5	0.0	4.0	.796

Table 4 illustrates demographic and clinical ER characteristics of study participants with ischemic ON (as current diagnosis on follow-up), according to their AION subtype (AAION or NAION). Significant associations were found in their diagnostic accuracy in the ER (p = 0.040) as AAION were all accurately diagnosed as ischemic, while NAION had a significative smaller prevalence (65.6%) of accurate diagnosis (65.6%, SR = -0.5). As distinguishing features between these two etiologies, AAION patients had more frequently bilateral eye involvement (33.3%, SR = 2.3) compared to NAION (3.1%) (p = 0.032), as well as a higher median age of onset (82 years vs 56 years) (p < 0.001) as can be seen in Table 5.

**Table 4.** Er demographic, clinical characteristics and exams performed in patients with ischemic ON at the end of follow-up.

Ischemic Optic Neuropathy category at end of follow-up							
	AAION		NAION		Total		
	n	% (SR)	n	% (SR)	n	%	p-value
Diagnostic accuracy							
Inaccurate	0	0.0%	5	15.6%	5	12.2%	.0207
Accurate	9	100.0%	21	65.6%	30	73.2%	
Unspecified etiology	0	0.0%	6	18.8%	6	14.6%	
ER Diagnosis							.040
Ischemic	9	100.0% (0.9)	21	65.6% (−0.5)	30	73.2%	
Non-Ischemic	0	0.0% (−1.6)	11	34.4% (0.8)	11	26.8%	
ER Specific diagnosis							<.001
Unspecified AION	0	0.0% (−1.3)	8	25.0% (0.7)	8	19.5%	
AAION	9	100.0% (5.0)	0	0.0% (−2.7)	9	22.0%	
NAION	0	0.0% (−1.7)	13	40.6% (0.9)	13	31.7%	
Inflammatory	0	0.0% (−1.0)	5	15.6% (0.6)	5	12.2%	
Unspecified ON	0	0.0% (−1.0)	6	18.8% (0.6)	6	14.6%	
Patient's gender							.147
Woman	3	33.3%	20	62.5%	23	56.1%	
Man	6	66.7%	12	37.5%	18	43.9%	
Unilateral or Bilateral							.032
One	6	66.7% (−0.7)	31	96.9% (0.4)	37	90.2%	
Bilateral	3	33.3% (2.3)	1	3.1% (−1.2)	4	9.8%	
Visual acuity - Affected Eye							.054
≤0.1	8	88.9%	15	46.9%	23	56.1%	
>0.1	1	11.1%	17	53.1%	18	43.9%	
Hypertension							.563
No	2	22.2%	12	37.5%	14	34.1%	
Yes	7	77.8%	20	62.5%	27	65.9%	
Dyslipidaemia							.334
No	2	22.2%	16	50.0%	18	43.9%	
Yes	7	77.8%	16	50.0%	23	56.1%	
Obstructive Sleep Apnea							>.990
No	9	100.0%	31	96.9%	40	97.6%	
Yes	0	0.0%	1	3.1%	1	2.4%	
Smoker							.563
No	5	55.6%	22	68.8%	27	65.9%	
Yes	4	44.4%	10	31.3%	14	34.1%	
Migraine							.179
No	9	100.0%	30	93.8%	39	95.1%	
Yes	0	0.0%	2	6.3%	2	4.9%	
Diabetes							.056
No	6	66.7%	29	90.6%	35	85.4%	
Yes	3	33.3%	3	9.4%	6	14.6%	
Weight Loss							>.990
No	8	88.9%	27	84.4%	35	85.4%	
Yes	1	11.1%	5	15.6%	6	14.6%	
Jaw claudication							>.990
No	9	100.0%	29	90.6%	38	92.7%	
Yes	0	0.0%	3	9.4%	3	7.3%	
Arthralgia							.815
No	8	88.9%	27	84.4%	35	85.4%	
Yes	1	11.1%	5	15.6%	6	14.6%	
Myalgia							>.990
No	9	100.0%	31	96.9%	40	97.6%	
Yes	0	0.0%	1	3.1%	1	2.4%	
Erythrocyte Sedimentation Rate							.408
Normal	4	44.4%	6	18.8%	10	24.4%	
Increased	0	0.0%	6	18.8%	6	14.6%	
Not performed	5	55.6%	20	62.5%	25	61.0%	
C-reactive protein							.906
Normal	3	33.3%	8	25.0%	11	26.8%	
Slightly increased	6	66.7%	19	59.4%	25	61.0%	
Not performed	0	0.0%	5	15.6%	5	12.2%	
Anaemia							.387
No	6	66.7%	21	65.6%	27	65.9%	
Yes	3	33.3%	6	18.8%	9	22.0%	
Not performed	0	0.0%	5	15.6%	5	12.2%	
Scalp Tenderness							>.990
No	8	88.9%	29	90.6%	37	90.2%	
Yes	1	11.1%	3	9.4%	4	9.8%	
Headache							.819
No	7	77.8%	21	65.6%	28	68.3%	
Yes	2	22.2%	11	34.4%	13	31.7%	
Electrocardiogram (ECG)							.789
Normal	0	0.0%	3	9.4%	3	7.3%	
Not performed	9	100.0%	29	90.6%	38	92.7%	
Performed Eco Doppler in ER							.060
No	9	100.0%	29	90.6%	38	92.7%	
Yes	0	0.0%	3	9.4%	3	7.3%	
Performed Eco Doppler in neurology ward							.570
No	7	77.8%	29	90.6%	36	87.8%	
Yes	2	22.2%	3	9.4%	5	12.2%	

Abbreviations: AAION, Arteritic Anterior Ischemic Optic Neuropathy; NAION, Non-arteritic Anterior Ischemic Optic Neuropathy.

**Table 5.** Associations between continuous clinical variables and ischemic ON diagnosis at the end of follow-up.

	AAION			NAION			
	Mdn	P25	P75	Mdn	P25	P75	p-value
Age at Admission	82.0	74.0	83.0	56.0	50.5	44.5	<.001
Interval between onset of symptoms and admission at emergency room (in days)	3.0	2.0	5.0	3.5	1.5	2.5	.504
Visual acuity – Affected Eye (10/10)	1.0	0.0	6.0	4.0	0.0	0.5	.256

Abbreviations: AAION, Arteritic Anterior Ischemic Optic Neuropathy; NAION, Non-arteritic Anterior Ischemic Optic Neuropathy.

We found also that 2 of the patients with Ischemic ON (4.8%) were being treated with a known neuritis inducing medication, namely amiodarone, prior to development of ON. Out of the 30 accurately diagnosed Ischemic ON in the ER, 8 diagnoses (26.7%) didn’t distinguish between AAION and NAION.

To determine predictors of diagnosis redefinition during follow-up, we analyzed possible predictive factors, comparing patients who had diagnostic reclassification to those who maintained their initial diagnosis category. The correspondent associations are shown in Table 6. Inflammatory diagnosis was more prevalent in the group with no diagnostic redefinition (72.8%, SR = 2.2) and less prevalent in group with diagnostic redefinition (17.4%, SR = -3.6). Significant association was found between follow- up diagnosis category and diagnostic redefinition (p = .005). Multiple sclerosis was associated with underrepresentation in diagnosis redefinition (10.9%, SR = -2.0). Other ON was associated with greater diagnosis redefinition (15.2%, SR = 2.3), when compared with the other groups.

**Table 6.** Performed exams during follow-up according to optic neuropathy diagnosis redefinition.

Optic Neuropathy Diagnostic Redefinition during follow-up							
	Yes		No		Total			
	n	% (SD)	n	% (SD)	n	%	p-value
Emergency room diagnosis category							<.001
Inflammatory	8	17.4% (−3.6)	91	72.8% (2.2)	99	57.9%	
Unspecified etiology	27	58.7% (7.3)	0	0.0% (−4.4)	27	15.8%	
Ischemic	8	17.4% (−0.7)	30	24.0% (0.4)	38	22.2%	
Other	3	6.5% (0.8)	4	3.2% (−0.5)	7	4.1%	
Actual diagnosis category							.005
Multiple sclerosis	5	10.9% (−2.0)	39	31.2% (1.2)	44	25.7%	
Idiopathic optic neuritis	17	37.0% (0.1)	44	35.2% (−0.1)	61	35.7%	
Other demyelinating/inflammatory	6	13.0% (1.2)	8	6.4% (−0.7)	14	8.2%	
Ischemic	11	23.9% (0.0)	30	24.0% (0.0)	41	24.0%	
Other optic neuropathy	7	15.2% (2.3)	4	3.2% (−1.4)	11	6.4%	
Performed Visual Evoked Response							.255
No	10	21.7%	40	32.0%	50	29.2%	
Yes	36	78.3%	85	68.0%	121	70.8%	
Performed Optical Coherence Tomography							.491
No	26	56.5%	62	49.6%	88	51.5%	
Yes	20	43.5%	63	50.4%	83	48.5%	
Blood: Immunology blood test - Findings							.815
No	36	78.3%	96	77.9%	132	79.0%	
Yes	7	15.2%	15	13.1%	22	13.2%	
Not performed	3	6.5%	10	9.0%	13	7.8%	
Blood: Microbiology test - Findings							.356
No	43	93.5%	110	89.5%	153	91.6%	
Yes	2	4.3%	12	10.5%	14	8.4%	
Performed Cerebrospinal fluid (CSF) analysis							.171
No	8	17.4%	35	28.0%	43	25.1%	
Yes	38	82.6%	90	72.0%	128	74.9%	
Performed anti-AQP4 test							.491
No	26	56.5%	62	49.6%	88	51.5%	
Yes	20	43.5%	63	50.4%	83	48.5%	
Aquaporin-4 antibodies Test							.629
Negative	20	43.5%	62	49.6%	82	48.0%	
Positive	0	0.0%	1	0.8%	1	0.6%	
Not performed	26	56.5%	62	49.6%	88	51.5%	
Performed anti-MOG test							.516
No	36	78.3%	86	72.2%	122	71.6%	
Yes	10	21.7%	38	30.8%	48	28.4%	
MOG Antibody Test							.164
Negative	8	17.4%	36	29.0%	44	25.9%	
Positive	2	4.3%	2	1.6%	4	2.4%	
Not performed	36	78.3%	86	69.4%	122	71.8%	
MRI: Brain - Demyelinating Lesions							.072
No	29	63.0%	70	56.0%	99	57.9%	
Yes	15	32.6%	33	26.4%	48	28.1%	
Null	2	4.3%	22	17.6%	24	14.0%	
MRI: Brain - Vascular Lesions							.043
No	39	84.8% (1.0)	84	67.2% (-0.6)	123	71.9%	
Yes	5	10.9% (-0.6)	19	15.2% (0.3)	24	14.0%	
Null	2	4.3% (-1.8)	22	17.6% (1.1)	24	14.0%	
MRI: Brain - Unspecified Lesions							.057
No	37	80.4%	82	65.6%	119	69.6%	
Yes	7	15.2%	21	16.8%	28	16.4%	
Null	2	4.3%	22	17.6%	24	14.0%	
MRI: Orbits							.389
Normal	20	44.5%	40	32.5%	60	35.7%	
Lesion	10	22.2%	32	26.0%	42	25.0%	
Not performed	15	33.3%	51	41.5%	66	39.3%	
MRI: Spinal Cord - Presence of lesions							.614
No lesions	7	15.2%	14	11.2%	21	12.3%	
Yes	5	10.9%	11	8.8%	16	9.4%	
Not performed	34	73.9%	100	80.0%	134	78.4%	

In the neurology ward, 121 patients performed VER (70.8%) and 83 performed OCT (48.5%). During neurology ward and posterior follow-up, 93 patients (54.4%) were screened for AQP4 antibodies, with 1 having a positive result (1.1%). Since the testing for MOG was introduced (in 2018), 49 patients searched for this antibody (33.3%) with 4 of those having a positive result (8.2%).

## Discussion

Disorders of the optic nerve, also known as optic neuropathies (ON), are among the most common causes of visual loss. They can occur independently or as a part of a wider neurological or systemic disorder, and typically manifest with decreased visual acuity, altered color vision and abnormal visual field in the affected eye. The causes of ON are diverse, and the diagnosis can often be suggested on the basis of patient's clinical history and clinical examination, of which several characteristics are particularly crucial to distinguish ON from retinal and ocular pathology. However, the precise diagnosis is challenging at the time of presentation, leading to delay in appropriate clinical management. As the prognosis and therapeutics approach can differ according to the mechanism involved, it is crucial to evaluate possible predictive factors that may influence the etiology identification. In the appropriate setting, directed evaluation with imaging studies and laboratory tests are important in establishing the correct diagnosis.

Our results showed that the overwhelming majority of patients (95.8%) with ON admitted to the neurology ward were sent from the ER, underlining the importance of having a high clinical suspicion for this condition in the emergency care setting. All participants, in our hospital, were diagnosed in the ER with suspicion of ON as a main diagnosis. Diagnostic inaccuracy appeared only when trying to classify the different pathophysiological causes of ON, which is consistent with the clinical consensus regarding the diagnosis of these diseases.^2,4^ Despite this fact, the great majority of patients (73.1%) was accurately classified regarding the etiological category, which we believe can be attributed to the evaluation of all patients by both a neurologist and an ophthalmologist, making this cooperation of crucial importance.

Other interesting finding of our data's analysis was that AAION are more accurately diagnosed as Ischemic ON in the ER when comparing to NAION, as well as puts forward clinical distinguishing features between them, namely a higher median age of onset in AAION, and a more frequent bilateral eye involvement consistent with previous descriptions.^13,14^

In accordance with the literature,^2,4^ our study demonstrated female dominance in almost all diagnostic categories. Although some authors claim that Non-Arteritic Ischemic ON (NAION) is more frequent in men, the female dominance in our population was 56.1%.

Regarding visual acuity, 56.1% of patients with Ischemic ON presented at admission with visual acuity ≤ 0.1. When compared AAION to NAION subtype, 88.9% of patients with AAION presented visual acuity ≤ 0.1, as would be expected. In what concerns eye pain, only 39.0% of patients diagnosed as Ischemic ON referred this symptom at admission. This result also meets the literature,^5,6,7–8^ which states that the absence of pain can help to differentiate Ischemic ON from other ON.

The reduced color vision is a very sensitive indicator of ON. The percentage of patients with dyschromatopsia was superior in MS-ON (29.5%) than those with Ischemic ON (22.0%). In what concerns fundoscopic findings, in our study, optic disk edema was the most frequent finding in all diagnoses, especially in Ischemic ON and other demyelinating/inflammatory ON.

The relative afferent pupillary defect (RAPD), as diagnosed by swinging flashlight test, is very evocative on an ON, whatever its etiology. Our results show that 74.2% of patients presented RAPD in the ER, which result supports the importance of a premeditated stepwise ophthalmological evaluation, as RAPD should be examined prior to the administration of a mydriatic for fundoscopic observation^4,15^

Toxic/nutritional ON are conditions that can present with similar symptoms, including progressive, symmetric central visual loss, decreased color vision, and optic disc pallor. The underlying mechanism of optic nerve injury is not well understood in most cases, and may involve multifactorial aspects. Among the medications associated to toxic ON, Amiodarone is an antiarrhythmic drug that has been associated with a bilateral or sequential optic neuropathy, mimicking Anterior Ischemic ON (AION). Amiodarone-associated ON was reported to occur in 1.76% of amiodarone user in 1987, by *Feiner et al*. Even in the modest sample of our population, we found two patients with Ischemic ON (4.8%) taking this medication, which reinforce the compelling relevance on searching for neuropathy-inducing medications in patients with suspicion of AION^15,16–17^

As previously suggested,^3,4,5–6^ our study demonstrated that diagnostic accuracy is mainly determined by medical history and neuro-ophthalmologic evaluation, as we found no statistically significative difference in diagnostic accuracy between patients who were submitted to imaging exams in the ER. While most patients performed imaging exams, overwhelmingly a head CT scan (79.0%), which undoubtedly has its clinical relevance in excluding neurological emergencies,^18,19–20^ our data shows that it will not contribute to further diagnostic accuracy regarding ON.

Advances in optic nerve imaging have enabled better visualization of the optic nerve. The OCT has become increasingly used in patients with ON, once that has permitted accurate documentation of retinal axonal degeneration, so as quantification of disc edema.^4,6,20,21^ We reported also that 14.7% of patients were examined by OCT since its use for ON in the ER began, reflecting the yet relatively small use in day- to-day practice in the emergency setting, despite its diagnostic capabilities.^11^ Furthermore, OCT is an objective way to detect even subclinical axonal and neuronal loss, and can facilitate early intervention and improved long-term visual outcomes.^21,22^

Additionally, this study demonstrates that patients with initial suspicion of ischemic ON are those in which reclassification is more likely to occur during follow-up. As distinguishing features between patients with actual ischemic ON and those misdiagnosed, we found that patients with ischemic ON had worse vision at presentation, higher prevalence of previous neurological events, painless acute or subacute visual loss and an older age of presentation. Taken together, these characteristics are usually evocative of Ischemic ON, which is compatible with clinical consensus,^4^ despite the fact that ischemic ON can present with normal visual acuity.^13^ In this population we also noted a low frequency of fundoscopic crowded disk evaluation in the contralateral eye of patients with a unilateral eye affected, which endangers vision loss on the contralateral eye. As previously suggested,^14,19^ this is a common occurrence in this particular etiology.

With a greater understanding of NMOSD and MOGAD, there has been an increasing reliance on the AQP4-IgG and MOG- IgG tests.^10,12,23,24^ Under suspected Neuromyelitis optica (NMO) or in cases with features atypical for MS-ON, testing for AQP4-IgG and MOG - IgG is advised, as the seropositivity has specific diagnostic and prognostic implications.

In our study, the follow-up data demonstrated that these antibody markers helped to reach a definitive etiological diagnosis in five patients, which was crucial for therapeutic decisions and follow-up.

The main limitations of our study are the modest sample size, and the fact that the population observed only comes from a single center. Additionally, the retrospective nature of this study can lead to inaccuracies or missing information on database between January 2004 and December 2021, which is an inevitable limitation of any study that goes back many years.

## Conclusions

This study describes the initial presentation and diagnosis in the ER room of the CHUSJ population hospitalized with ON in the last 18 years and it analyses the diagnostic accuracy at the onset of disease according with the patient's most recent diagnosis. Furthermore, we analyzed clinical characteristics of different ON etiological groups, characterizing our cohort and illustrating that most patients with ON can be accurately diagnosed in the ER mainly with medical history and neurological and ophthalmological evaluation. As soon as the diagnostic is stated, the disease-specific therapeutic can be initiated, minimizing injury and improving recovery. Additionally, it is demonstrated that patients with initial suspicion of Ischemic ON are those in which reclassification is more likely to occur during follow-up. In what concerns clinical findings that may reinforce diagnostic accuracy, we suggest: vision loss <0.1, previous neurological events, and age of onset. We also describe a lower diagnosis accuracy of NAION as Ischemic ON compared to AAION. We recognize the need for further research to validate our results.
